# Recent Advances in Immunotherapy for Bladder Cancer Treatment

**DOI:** 10.7759/cureus.79002

**Published:** 2025-02-14

**Authors:** Khaled A Alqarni

**Affiliations:** 1 Department of Surgery, Faculty of Medicine, University of Jeddah, Jeddah, SAU

**Keywords:** atezolizumab, bladder cancer, immunotherapy, nivolumab, urothelial carcinoma

## Abstract

Bladder cancer is one of the most common malignancies worldwide. Standard neoadjuvant or metastatic therapy used to be cisplatin-based chemotherapy, but many patients are ineligible due to age, renal impairment, or frailty. Checkpoint inhibitors (e.g., atezolizumab and pembrolizumab) enhance survival in cisplatin-ineligible patients. Originally approved as second-line therapy for patients after platinum-based chemotherapy, nivolumab was approved by the FDA for adjuvant therapy of high-risk muscle-invasive urothelial cancer following the Checkmate 274 trial. It is indicated for patients with resected disease or cisplatin ineligibility. Recent developments focused on the contribution of nivolumab to outcomes have been complemented by ongoing investigations on atezolizumab as a monotherapy or in combinations for muscle-invasive bladder cancer, providing further hope for improved control.

This narrative review aims to clarify the current applications of immunotherapy in treating bladder cancer and to explore the future outlook based on ongoing clinical trials.

## Introduction and background

Around 200,000 people die each year from bladder cancer, the 12th most common malignancy in the world [1,2]. According to studies, men are more likely to develop bladder cancer [3]. Transurethral resections are a standard treatment for superficial bladder cancer. However, most cases have a high recurrence rate and are not muscle-invasive [4]. When diagnosed, over 75% of bladder cancer patients had non-muscle-invasive bladder cancer (NMIBC). These can be successfully treated using conservative local treatments and careful surveillance [4].

Twenty-five percent have muscle-invasive disease, typically requiring cystectomy, radiotherapy, or palliative treatment [5,6]. Muscle-invasive bladder cancer (MIBC), on the other hand, is a more aggressive form of bladder cancer that requires local treatments like radical cystectomy and radio chemotherapy to achieve a curative outcome [7]. Localized MIBC patients have a survival rate of 70% over five years. Patients with lymph node involvement have a 39% survival rate [8]. Patients with distant metastases, however, have an 8.8% survival rate [9].

Urothelial carcinoma (UC) is responsive to chemotherapy and can be administered alongside radical cystectomy. It serves as neoadjuvant treatment, offering a 5% increase in survival rates, or as adjuvant therapy for localized disease or lymph node involvement, which provides a 10% survival advantage [7]. Approximately 40% of patients experience recurrence or metastasis following radical cystectomy [10,11]. Furthermore, 10-15% of UC patients present with metastatic disease at diagnosis [12]. In the absence of treatment, the median survival for metastatic UC is only three to six months [13]. By the time of death, nearly all UC patients show extensive and often symptomatic metastasis [12].

Our immune system can identify and destroy any potentially harmful cells. T cells are specifically trained to search our bodies for malignant cells. Most advanced cancer patients do not develop anti-cancer immunity or are suppressed due to the cancer’s malignant growth. Cancer cells use various methods to bypass and weaken our defenses. Cancer cells’ remarkable ability to hide from the immune system and rewire after treatment has been challenging for cancer treatments. Hacking the immune system to reprogram immune cells so they react to cancerous cells is one way to fight cancer. Recently, a novel family of monoclonal antibodies that increase T cell activity was identified. This improves the cytotoxic reaction and tumor cell death [14]. Immunotherapy may be used to train the body’s immune system and help it fight cancer. Immunotherapy or biological therapy aims to increase the immune system’s ability to eliminate cancer cells. Immune checkpoints are a group of immune system inhibitory pathways designed to manage physiological immune reactions in tissues and limit or prevent collateral tissue injury. Two such immune checkpoints that have garnered particular interest for cancer immunotherapy treatment are programmed cell death protein 1 (PD-1) and cytotoxic T-lymphocyte-associated antigen 4 (CTLA-4); two highly popular immunological checkpoints are CTLA-4 and CD152, respectively. These two immune systems’ inhibitory checkpoints are the focus of drug development for cancer [14].

Through bacillus Calmette-Guérin (BCG) therapy and immune checkpoint inhibitors, immunotherapy has become a crucial treatment method for bladder cancer. BCG, which is a live attenuated *Mycobacterium bovis*, has been used for the treatment of NMIBC since the 1970s. When applied inside the bladder, it elicits a local immune response that destroys bladder cancer cells. It is thought that the process engages both innate and adaptive immune systems, which include T-cells and natural killer (NK) cells that play an important role in the eradication of tumors [15].

BCG therapy is especially useful in patients with high-risk NMIBC because it not only prevents the recurrence of tumors but also delays the development of muscle-invasive disease [16,17]. The response to BCG is quite strong; it initiates an immune response that leads to the accumulation of immune cells in the tumor microenvironment, which is vital for the response. About 30-50% of patients do not respond well to BCG. Hence, predictive biomarkers are needed to identify patients who will benefit from this treatment [18].

Systemic immunotherapy has also shown its usefulness in treating advanced bladder cancer in addition to BCG. Checkpoint inhibitors such as PD-1 and programmed death-ligand 1 (PD-L1) inhibitors have been licensed for use in advanced and metastatic UC. They function by disrupting the mechanisms through which tumors disable the immune system and thus allow the immune system to recognize and attack the cancer cells [19,20]. The response to these treatments depends on the tumor microenvironment and the levels of particular immune cells that can differ from one patient to another [20,21].

Current investigations have also focused on tumor-specific T cells and different biomarkers for response prediction in immunotherapy. For instance, the levels of PD-L1 have been linked with improved survival when patients are on checkpoint inhibitors [22].

However, the challenges include identifying patients who will benefit most from the treatment and managing the side effects associated with these therapies. Bladder cancer is a complex immune system disease that requires a combination of treatment approaches, including immunotherapy with chemotherapy and/or targeted therapies to enhance the response [23].

This narrative review aims to clarify the current applications of immunotherapy in treating bladder cancer and to explore the future outlook based on ongoing clinical trials.

## Review

Immune checkpoint therapy in the treatment of bladder cancer

Oncology patient care has long relied on surgery, chemotherapy, radiotherapy, and targeted therapies to target cancer cells directly. Immune checkpoint therapies are a new and exciting option that uses the immune system of patients to target cancer specifically. While research is focused on finding cures for cancers of all types, combination methods can produce robust responses that last decades.

The gold standard for treating bladder cancer in its neoadjuvant or metastatic stages has been systemic chemotherapy regimens based on cisplatin until recently. Von der Maase et al. state that people who receive platinum-based chemotherapy for metastatic or locally advanced disease typically live nine to 15 months on average [24]. Checkpoint inhibitors are changing the treatment paradigms of urothelial cancers [25]. Immune checkpoint inhibitors are gaining tremendous interest in their potential as therapeutic agents for solid tumors.

These drugs target inhibitory receptors or ligands found on T lymphocytes and tumor cells to restore the optimal function of anti-cancer immunity. Immune checkpoint inhibitors are now an essential part of bladder cancer treatment to increase overall survival and decrease the morbidity associated with it. They also help maximize overall mortality and survival rates while simultaneously reducing morbidity. For example, nivolumab is an effective option for adjuvant therapies following neo-adjuvant platinum-based chemotherapy or if ineligible for chemotherapy. Pembrolizumab and enfortumab vedotin are used as first-line therapy for patients with metastatic disease [26].

Cancer cells are difficult for the immune system to identify and eliminate. In theory, cancer cells should be eliminated by the immune system when they decrease antigen expression or change their microenvironment. However, malignancies that survive can avoid immune system reactions and continue to live. Tumor cells attract immunosuppressive cell types like regulatory T-cells (Tregs), myeloid suppressor cells, and others. They then release substances that further contribute to the cancer [27].

Tregs can inhibit tumor-specific lymphocytes through the secretion of PD-L1, which attaches itself to receptors on T cells like PD-1 (B71) or CD80. This sends an inhibitory message and renders them ineffective [28]. Antitumor responses are strengthened, and fatigued T-cells are relieved from their burden when this binding between PD-L1 & PD-1 is blocked. Some examples of drugs that block the binding between these proteins are monoclonal antibodies against PD-L1, including atezolizumab, durvalumab, avelumab, nivolumab, and pembrolizumab. Monoclonal antibodies targeting CTLA-4 (another T cell regulatory mechanism) have led to the creation of ipilimumab, tremelimumab, and other drugs. CTLA-4 levels can be increased to compete with CD80/CD86 and prevent intracellular signals when T cells are activated. This reduces the immune response. CTLA-4 can be inhibited or restricted to prevent intracellular signaling. This reduces immune response.

Type of treatment

PD-L1 Inhibitors

**Figure 1:**
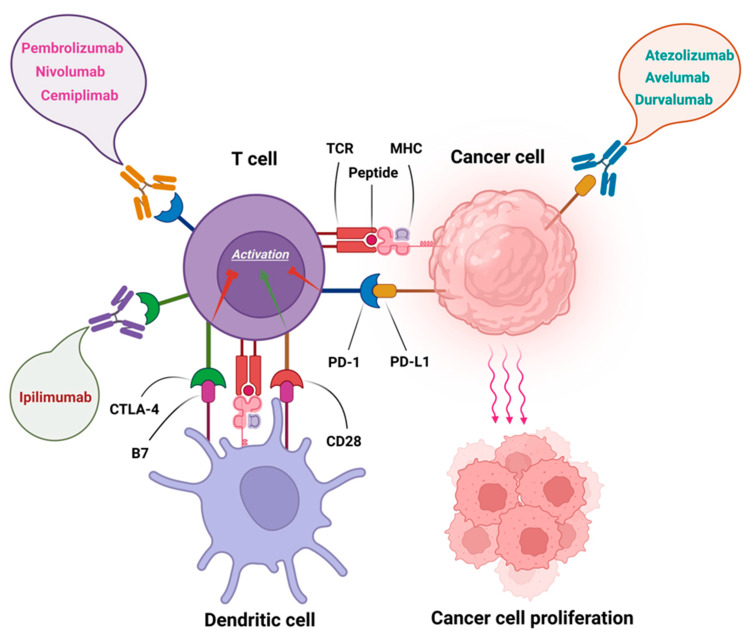
Inhibitors of immune checkpoints. Anti-PD-1 antibodies pembrolizumab, nivolumab, and cemiplimab, anti-CTLA-4 antibody ipilimumab, and anti-PD-L1 antibodies atezolizumab, avelumab, and durvalumab. Source: Shiravand et al. [29]. Used under the Creative Commons CC BY license. No special permission is required to reuse all or part of an article published by MDPI, including figures and tables. TCR: T-cell receptor; MHC: major histocompatibility complex; CTLA-4: cytotoxic T-lymphocyte-associated antigen 4; CD28: cluster of differentiation 28; PD-1: programmed cell death protein 1; PD-L1: programmed death-ligand 1.

Atezolizumab: Atezolizumab is a humanized monoclonal antibody that acts as an immune checkpoint inhibitor by binding to PD-1 receptors and blocking communication with B7-1 receptors. In May 2016, the FDA granted atezolizumab expedited approval for patients who did not respond to chemotherapy, such as cisplatin-based therapy. This lack of response could be due to the treatment being ineffective or progression occurring within a year after adjuvant or post-adjuvant platinum treatments, or it could be that the patients were chemotherapy-naive and ineligible for cisplatin treatment [27-30]. Typically, 1200 mg of atezolizumab is administered subcutaneously every three weeks. The IMVigor 210 trial, a phase II multicenter non-randomized study, was groundbreaking in demonstrating the efficacy and safety of atezolizumab for treating urothelial cancer [31]. In this study, 119 patients who were ineligible for cisplatin-based chemotherapy had an overall response rate (ORR) of 24%, observed across all subgroups of PD-L1 in tumor-infiltrating immune cells. In comparison, only 15% of the 316 patients who received cisplatin chemotherapy achieved an ORR. However, higher expression of PD-L1 (>5%) correlated with a higher ORR (23%) and longer median progression-free survival (PFS) (11.4 months versus 2.1 months) (Figure 1) [32].

Durvalumab: Durvalumab, a monoclonal anti-PD-L1 antibody, prevents it from attaching to PD-1. The dose should be 10 mg/kg intravenously given every two weeks until there is progress or excessive toxicities. The FDA approved durvalumab in May 2016 for metastatic urothelial cancers that were inoperable or had progressed after platinum-based treatment. The FDA granted approval based on phase I/II open-label multicenter research that included patients with metastatic solid tumors [33]. Durvalumab demonstrated an ORR of 61 patients across three phase 1/2 trials [33]. Responses were more significant (46%) for groups with high PD-L1 expression (defined as 25% positive tumor or infiltrating cell) compared to none in low expression levels.

Avelumab: Avelumab is another monoclonal anti-cancer antibody that binds to PD-1, blocking the interaction between PD-L1 and PD-1, thereby preventing adhesion. Avelumab is an FDA-approved cancer medication to treat localized or distantly advanced UC. The dosage is 10 mg/kg intravenously given every two weeks if the side effects or condition persists. The JAVELIN Solid Tumor Study approved avelumab in patients with local or metastatic urothelial cancer. Avelumab has been evaluated in its ongoing phase 1a dose-escalation study for safety, tolerability, and efficacy against solid tumor patients with metastatic or localized advanced disease [34].

PD-1 Inhibitors

Nivolumab: The FDA has approved nivolumab as a treatment for advanced urothelial cancer that has spread regionally or locally. Patients who received platinum-based chemotherapy for metastatic or locally advanced urothelial cancer were included in a study demonstrating the efficacy and safety of nivolumab. CheckMate 032 is a multicenter phase 1/2 open-label study that evaluated the safety and efficacy of nivolumab in two stages with multiple arms [35]. Checkmate 275, a phase 2 multicenter single-arm trial, evaluated nivolumab. This added more evidence. The trial involved patients with metastatic or surgically irresectable urothelial cancer who had received platinum-based chemotherapy [36].

Pembrolizumab: Pembrolizumab is a monoclonal humanized antibody that blocks PD-1. This transmembrane is expressed in T cells and natural killer cells. Pembrolizumab has been shown to have promising results in bladder cancer trials, with an ORR of 25-38% (KEYNOTE-012) for phase 1 studies. At least one patient had high PD-1 levels >1% tumor nests.

The phase II trial (KEYNOTE-042) intermediate analysis revealed an ORR of 36.7% for all patients and 24.0% in those with 10% positive PD-L1 expression. In phase III research, the median overall survival of second-line therapy compared to chemotherapy was 10.3 vs. 7.4 months [37,38].

CTLA-4 Inhibitors

Ipilimumab: Ipilimumab is a fully human monoclonal antibody that blocks CTLA-4. Although CTLA-4 inhibitors are not yet approved as treatments for bladder cancer, preliminary studies have demonstrated that they can help T cells recognize tumor antigens more effectively in individuals with urothelial cancer [39].

Clinical trials

Immunotherapy for the Treatment of Non-muscle Invasive Bladder Cancer

A phase 2 trial was conducted across 54 sites in a single-arm study called KEYNOTE-057. The research focused on patients in cohort B who were at least 18 years old, had an Eastern Cooperative Oncology Group (ECOG) performance status between 0 and 2, and had high-risk non-muscle-invasive bladder cancer that was resistant to BCG treatment. Patients were eligible for the study if they had high-grade Ta papillary tumors or any grade T1 tumors and showed no evidence of carcinoma in situ. Pembrolizumab 200 mg is given as an intravenous infusion every three weeks for a maximum of 35 treatment cycles. The 12-month disease-free survival rate was estimated at 43.5% (95% CI: 34.9-51.9%). Across the cohort, 97 patients (73%) experienced treatment-related adverse events; 19 patients (14%) developed grade 3 or 4 adverse events. The findings reveal that pembrolizumab monotherapy showed effective tumor control with reasonable safety for patients with BCG-unresponsive high-risk Ta or T1 bladder cancer without carcinoma in situ [40].

Immunotherapy for the Treatment of Locally Advanced or Metastatic Bladder Cancer

The IMvigor211 trial was a multicenter phase 3 randomized controlled study evaluating the efficacy of atezolizumab compared with chemotherapy among patients with platinum-refractory metastatic UC. Conducted across 217 sites, the trial included 931 patients who were randomized (1:1) to receive atezolizumab (1200 mg every three weeks) or chemotherapy (physician’s choice of vinflunine, paclitaxel, or docetaxel). In this sample population (n = 234), overall survival (OS) was reported to be not significantly different between the atezolizumab and chemotherapy groups (median OS: 11.1 months vs. 10.6 months) and a hazard ratio of 0.87, precluding further statistical analyses. Confirmed response rates were comparable (23% vs. 22%), though response duration was numerically longer from atezolizumab (median: 15.9 months vs. 8.3 months; HR: 0.57; 95% CI: 0.26-1.26). In the aim-to-treat patients, atezolizumab demonstrated a more favorable safety profile, with fewer grade 3-4 treatment-related adverse events (20% vs. 43%) and fewer treatment discontinuations due to adverse effects (7% vs. 18%). While atezolizumab did not confer a significant OS benefit over chemotherapy in PD-L1-overexpressing tumors, it was associated with durable responses and improved tolerability. These findings support its potential role as a treatment option for metastatic UC, particularly in patients prioritizing safety and quality of life [41].

A phase 3, randomized controlled trial was performed to analyze the efficacy of maintenance avelumab plus best supportive care (BSC) versus BSC alone in patients with unresectable locally advanced or metastatic UC who did not experience disease progression after first-line platinum-based chemotherapy. A total of 700 patients were assigned randomly in a 1:1 ratio to receive either maintenance avelumab or BSC alone. The results demonstrated a significant OS benefit with avelumab; at one year, OS was 71.3% among the avelumab group versus 58.4% in the comparative group, i.e., the control group (median OS: 21.4 vs. 14.3 months; HR for the death: 0.69). In the PD-L1-positive subgroup, OS at one year was 79.1% among avelumab versus 60.4% with BSC alone (HR: 0.56). PFS was also improved, with a median of 3.7 months in the avelumab group and 2.0 months in the control group (HR: 0.62), and 5.7 months versus 2.1 months in the PD-L1-positive subgroup (HR: 0.56). The adverse events of any complexity occurred in 98.0% of avelumab-treated patients and 77.7% of those in the control group. These findings establish maintenance avelumab as a standard-of-care option for patients with advanced UC whose disease has not progressed following first-line platinum-based chemotherapy, demonstrating a significant survival benefit without introducing severe toxicity [42].

A phase 3, randomized controlled trial (LEAP-011) was conducted to evaluate the efficacy along with the safety of pembrolizumab plus lenvatinib versus pembrolizumab plus placebo as first-line therapy in patients with advanced UC who were not given cisplatin-based or any platinum-based chemotherapy. The research assigned 487 patients for this trial. During the 12.8-month median follow-up period, the combination arm registered a median PFS of 4.5 months while the pembrolizumab-alone arm reported 4.0 months as the PFS (HR: 0.90). Patients in both trial groups experienced an estimated survival period of 11.8 months for the combination therapy and 12.9 months for pembrolizumab monotherapy at a patient follow-up rate of 12.8 months (HR: 1.14). Adverse events of grade 3-5 severity manifested in treatment cycles for 51% of patients receiving combination therapy but only affected 27% of patients in the pembrolizumab-alone group. The trial halted its planned course of action because the data monitoring committee pronounced enough evidence showing no clinical advantage while noting enhanced adverse events. Pembrolizumab monotherapy stands as the preferred initial therapy when platinum-based chemotherapy is not suitable for patients with advanced UC [43].

Immunotherapy for the Treatment of Muscle-Invasive Bladder Cancer as a Neoadjuvant

The ABACUS trial was a multicenter, phase 2 study assessing the efficacy of neoadjuvant atezolizumab in patients with MIBC who were not eligible for or declined cisplatin-based chemotherapy. A total of 95 patients received at least one cycle of atezolizumab prior to radical cystectomy, with serial blood and tissue samples collected for biomarker analysis. The study’s primary endpoints, including pathological complete response (pCR) and T-cell biomarker changes, were previously reported, while this analysis focused on secondary endpoints of two-year disease-free survival (DFS) and OS, along with exploratory biomarker correlations. After a median follow-up of 25 months, the pCR rate was 31%. The estimated two-year DFS and OS were 68% and 77%, respectively, with DFS reaching 85% among patients achieving pCR. Biomarker analysis revealed that baseline PD-L1 expression and tumor mutational burden were not significantly correlated with relapse-free survival (RFS). However, high baseline stromal CD8+ expression (HR: 0.25) and elevated post-treatment fibroblast activation protein (HR: 4.1) were significantly associated with RFS. Circulating tumor DNA (ctDNA) analysis showed progressive reductions in positivity rates from baseline (63%), after neoadjuvant therapy (47%), to post surgery (14%), with ctDNA negativity. Despite its single-arm design and exploratory biomarker analysis, the study demonstrates that neoadjuvant atezolizumab is associated with favorable clinical outcomes in MIBC. Furthermore, CD8+ expression and serial ctDNA assessments may serve as valuable predictive biomarkers, potentially guiding future personalized treatment strategies [44].

A phase 3 placebo-controlled randomized trial was conducted to investigate the efficacy of perioperative immunotherapy in MIBC. A total of 1063 cisplatin-eligible patients were randomly added to the durvalumab group, who received neoadjuvant durvalumab and gemcitabine and cisplatin, radical cystectomy, and then durvalumab adjuvant versus comparison to the neoadjuvant gemcitabine and cisplatin with radical cystectomy alone. In the durvalumab group, findings observed a marked improvement in event-free survival at 24 months compared to the comparison group (67.8% vs. 59.8%, HR: 0.68). As in the initial analysis, overall survival at 24 months was higher in the durvalumab group (82.2%) than in the comparison group (75.2%) (HR for death: 0.75). In addition, 88.0% of patients treated with durvalumab compared to 83.2% of patients in the comparison received radical cystectomy. Therefore, perioperative durvalumab might improve survival with tolerable toxicity when used in combination with standard neoadjuvant chemotherapy in MIBC or as an adjuvant treatment strategy [45].

Future perspective

In the next five to 10 years, advancements in precision medicine and biomarker-driven trials will likely enable the personalized application of immunotherapies, optimizing efficacy while minimizing adverse effects in UC treatment. Combination approaches integrating checkpoint inhibitors with novel immunomodulators, targeted therapies, and even mRNA-based cancer vaccines may redefine standards of care for both early-stage and metastatic bladder cancers. Additionally, the emergence of advanced diagnostic tools and artificial intelligence could significantly enhance the prediction of patient responses, leading to more efficient, individualized treatment plans.

## Conclusions

This impasse is addressed by the discovery of immune checkpoint inhibitors that specifically target the PD-1/PD-L1 pathway. This breakthrough is set to transform the conventional understanding of advanced carcinoma treatment. Bladder cancer research is particularly thrilling at this time due to the antitumor efficacy and tolerability of these therapies. These promising advancements may offer hope to individuals with bladder urothelial cancer. Ongoing research seeks to identify the most effective immunotherapy regimens for treating urothelial carcinoma in both neoadjuvant and adjuvant settings, as well as in metastatic cases. Future studies are required to explore mono- or combination checkpoint inhibitor therapy and biomarker-driven approaches for treating bladder cancers.
